# The Low FODMAP Diet: Many Question Marks for a Catchy Acronym

**DOI:** 10.3390/nu9030292

**Published:** 2017-03-16

**Authors:** Giulia Catassi, Elena Lionetti, Simona Gatti, Carlo Catassi

**Affiliations:** Department of Pediatrics, Università Politecnica delle Marche, Via F. Corridoni 11, 60123 Ancona, Italy; giulia.catassi@gmail.com (G.C.); mariaelenalionetti@gmail.com (E.L.); simona.gatti@hotmail.it (S.G.)

**Keywords:** low-FODMAP diet, irritable bowel syndrome, non-celiac gluten sensitivity, fermentable sugars, polyols, nutritional risk

## Abstract

FODMAP, “Fermentable Oligo-, Di- and Mono-saccharides And Polyols”, is a heterogeneous group of highly fermentable but poorly absorbed short-chain carbohydrates and polyols. Dietary FODMAPs might exacerbate intestinal symptoms by increasing small intestinal water volume, colonic gas production, and intestinal motility. In recent years the low-FODMAP diet for treatment of irritable bowel syndrome (IBS) has gained increasing popularity. In the present review we aim to summarize the physiological, clinical, and nutritional issues, suggesting caution in the prolonged use of this dietary treatment on the basis of the existing literature. The criteria for inclusion in the FODMAPs list are not fully defined. Although the low-FODMAP diet can have a positive impact on the symptoms of IBS, particularly bloating and diarrhea, the quality of the evidence is lower than optimal, due to frequent methodological flaws, particularly lack of a proper control group and/or lack of blinding. In particular, it remains to be proven whether this regimen is superior to conventional IBS diets. The drastic reduction of FODMAP intake has physiological consequences, e.g., on the intestinal microbiome and colonocyte metabolism, which are still poorly understood. A low-FODMAP diet imposes an important restriction of dietary choices due to the elimination of some staple foods, such as wheat derivatives, lactose-containing dairy products, many vegetables and pulses, and several types of fruits. For this reason, patients may be at risk of reduced intake of fiber, calcium, iron, zinc, folate, B and D vitamins, and natural antioxidants. The nutritional risk of the low-FODMAP diet may be higher in persons with limited access to the expensive, alternative dietary items included in the low-FODMAP diet.

## 1. Introduction

FODMAP, “Fermentable Oligo-, Di- and Mono-saccharides And Polyols”, is a heterogeneous group of highly fermentable but poorly absorbed short-chain carbohydrates and polyols. The acronym FODMAP was first coined in 2005 by Gibson and Shepherd at Monash University in Melbourne, Australia, in a personal view article suggesting a link between the western lifestyle, the intake of FODMAP-rich foods, and susceptibility to Crohn's disease [1]. Soon after, the Australian group focused on the use of a low-FODMAP diet in the treatment of irritable bowel syndrome (IBS) [2], and in one of their most influential papers they showed that symptom improvement in patients with IBS and suspected non-celiac gluten sensitivity (NCGS) was not related to gluten avoidance, but to the concomitant reduction of FODMAP intake determined by the gluten-free diet (GFD). Interestingly, that study was double-blinded and placebo-controlled for the gluten challenge, but not for the reduction of dietary FODMAPs [3]. In recent years the putative role of FODMAPs in IBS has gained wide popularity in the general public and the subject has been addressed in books promoting the low-FODMAP diet and related recipes [4]. The recently revised British Dietetic Association (BDA) guidelines for the dietary management of IBS recommend the low-FODMAP diet as the second-line intervention in IBS patients [5]. A 2016 meta-analysis supports the efficacy of a low-FODMAP diet in the treatment of functional gastrointestinal symptoms [6]. Treatment with the low-FODMAP diet has also been advocated for diverticulitis [7], exercise-induced gastrointestinal symptoms [8], and inflammatory bowel diseases [9]. Although these studies indicate that a subgroup of patients with IBS may benefit from eating less highly fermentable sugars, i.e., the low-FODMAP diet [6], there are still several open questions regarding the physiology, the efficacy, and the safety of this dietary treatment.

In this paper the physiological, clinical, and nutritional issues suggesting caution in the prolonged use of the low-FODMAP diet will be summarized on the basis of the existing literature. The literature search was conducted in the PubMed MEDLINE and SCOPUS databases using the term “FODMAP” and “irritable bowel syndrome”, and only articles in English were extracted. We identified 98 papers, and selected 17 prospective, intervention trials for analysis.

## 2. What Is FODMAP?

FODMAP is not a single entity, but a group of compounds, including oligosaccharides (fructans, fructo-oligosaccharides = FOS and galacto-oligosaccharides = GOS), disaccharides (lactose), monosaccharides (fructose), and polyols (sorbitol, mannitol, maltitol, xylitol, polydextrose, and isomalt). The list of dietary sugar alcohols (polyols) includes tens of compounds used widely and unpredictably by the food industry as thickeners and sweeteners. Lactose belongs to FODMAPs only in individuals showing non-persistence of high lactase levels, which is a highly variable percentage of subjects in different populations. On the other hand, lactulose is an orally administered, non-absorbable disaccharide that is used in the treatment of constipation, a problem affecting many patients with IBS, and should definitely be avoided in subjects undergoing the FODMAP exclusion.

The FODMAP definition is based on functional instead of biochemical characteristics: being poorly absorbable and highly fermentable in the intestine is the common denominator of FODMAPs. They might exacerbate IBS symptoms through various mechanisms, such as increasing small intestinal water volume, colonic gas production, and intestinal motility. Conversely, FODMAPs have important physiological effects: they increase stool bulk, enhance calcium absorption and modulate immune function, and decrease the levels of serum cholesterol, triacylglycerols, and phospholipids. They selectively stimulate the growth of some microbial groups such as *Bifidobacteria* (prebiotic effect) [10]. Due to their capacity to stimulate the growth of nonpathogenic intestinal microflora, FOS and GOS are increasingly included in food products and infant formulas [11]. Fermentation of small, fermentable carbohydrates in the colon results in the production of short-chain fatty acids (SCFAs = acetate, propionate, and butyrate) that have a trophic effect on the colonocyte metabolism by increasing energy production and cell proliferation, and protecting against colon cancer [12,13]. All of the above positive effects are obviously lost with the low-FODMAP diet.

The boundaries of a low-FODMAP diet are not perfectly known. The appreciable work of the Melbourne group produced some analytical tables on the food content of specific FODMAPs [14,15,16], however, (a) many commercial items are missing in this list; and (b) the content of FODMAPs in vegetables is highly variable, e.g., according to the degree of maturation [17]. Furthermore, the possible interactions between FODMAPs and other nutrients are still unclear.

Finally, how much is a “normal” and how much is a low FODMAP intake? This has not yet been defined in quantitative terms.

## 3. Efficacy of the Low-FODMAP Diet: What Is the Quality of the Evidence?

Table 1 reports the clinical trials that are available in the literature on the effect of a low-FODMAP diet in IBS patients [3,18,19,20,21,22,23,24,25,26,27,28,29,30,31,32,33]. In general, most studies and one meta-analysis [6] have shown that IBS symptoms, particularly bloating and abdominal pain, may benefit from this treatment. However, the quality of the evidence is lower than optimal in our opinion, due to frequent methodological flaws, particularly a lack of a proper control group and/or lack of blinding, as shown in the last column of Table 1. The finding that the low-FODMAP diet improves IBS symptoms in comparison with a normal diet does not prove that this treatment is superior to the conventional IBS dietary intervention, e.g., the restriction of high-fiber food, resistant starch, fresh fruit, coffee, tea, alcohol, fizzy drinks and sorbitol, as recommended by the British National Institute for Health and Care Excellence (NICE) guidelines [34].

Indeed, studies comparing the efficacy of the low-FODMAP diet vs. proper dietary advice for IBS did not show a clear-cut advantage of the low-FODMAP diet: (a) in a US trial, 40%–50% of patients reported adequate relief of their IBS with diarrhea symptoms with the low-FODMAP diet or a diet based on modified NICE guidelines, even though the low-FODMAP diet led to significantly greater improvement in individual IBS symptoms, particularly pain and bloating, compared with the NICE diet [31]; (b) in a Swedish study the severity of IBS symptoms was reduced in both the low-FODMAP and the conventional IBS diet groups, at the end of a four-week period of treatment [26].

Due to the lack of a biomarker of “FODMAP intolerance”, the gold standard for proving the causal role of FODMAP, as well as other food intolerances/allergies, remains the double-blind placebo-controlled (DBPC) challenge. Three FODMAP challenge studies showed that high doses of fructose or fructans significantly worsen IBS symptoms [18,32,33]. Another randomized, DBPC study showed that gastrointestinal symptoms increased significantly after sorbitol and mannitol ingestion in patients with IBS compared to controls [14]. No DBPC challenge study is available for other FODMAPs or a mixed FODMAP-containing diet.

The duration of treatment with the low-FODMAP diet is rather short in the majority of published studies. This is a limitation for the evaluation of the low-FODMAP diet’s long-term efficacy. IBS is a chronic/recurrent condition but this treatment is difficult to maintain over time, due to many food exclusions. In a recent follow-up study of patients with IBS or inflammatory bowel disorder (IBD) treated with the low-FODMAPs diet, only one-third were still adherent to the diet after a median follow-up of 18 months [35]. The inventors of the low-FODMAP diet suggest an “all-FODMAP” free diet for two months followed by a serial challenge with one FODMAP per week (so called FODMAP reintroduction plan) [4]. Not only is the rationale of this challenge unclear, given that the physiological effects of FODMAP are not expected to change in such a short period of time, but also unpractical, since the list of food to reintroduce on a weekly basis is extremely long.

## 4. Is a Low-FODMAP Diet a Safe Approach?

The drastic reduction of FODMAP intake could have consequences that are still poorly understood on (a) the colonocyte metabolism (see Paragraph 2), (b) the intestinal microbiota, and (c) the nutritional status.

There is good evidence supporting the concept that the intestinal microbiota is perturbed in patients with IBS. Several recent studies have reported an increase in the relative abundance of Firmicutes, mainly *Clostridium* cluster XIVa, and Ruminococcaceae, together with a reduction in the relative abundance of *Bifidobacteria*. A lower diversity and a higher instability of the microbiota in IBS patients compared to controls have also been reported [36]. A low-FODMAP diet paradoxically does not correct these microbiota modifications, but induces similar changes, i.e., reducing the *Bifidobacteria* counts [20], and the total bacterial abundance [22], while increasing the abundance of Ruminococcacae [36]. This dietary treatment induces decreased levels of fecal *Faecalibacterium prausnitzii* and total SCFAs/n-butyric acid [33]. Extensive analyses of microbiota composition, functionality, and fermentation products in relation to symptom generation are currently lacking. Finally, the long-term effects of the microbiota changes induced by the low-FODMAP diet, if any, remain to be determined.

A low-FODMAP diet imposes an important restriction of dietary choices due to the elimination of some staple foods, such as wheat derivatives, lactose-containing dairy products, many vegetables and pulses, and several types of fruits (Table 2).

Despite the lack of studies on the long-term nutritional consequences of the low-FODMAP diet, possible risks of this treatment may be inferred from data available for other exclusion diets. As for cereal intake, the exclusion of wheat, rye, and barley is the same as the gluten-free diet (GFD) used for celiac disease treatment. Nutritional surveys have shown that subjects on a GFD may be at risk of reduced intake of fiber, calcium, iron, zinc, folate, and other B-group vitamins [37]. A deficient intake of dietary fiber may be expected to occur even more frequently on the low-FODMAP diet, due to a significant restriction of other fiber sources, such as fruit, vegetables, and legumes. The consequences of a fiber-poor diet may be particularly deleterious in subjects complaining of constipation as a manifestation of IBS. The restriction of lactose-containing dairy products may enhance the tendency to poor calcium availability since (a) these items are a primary source of calcium; and (b) the promoting effect of lactose on calcium absorption is lost [38,39]. A low-FODMAP diet may also be poor in natural antioxidants, such as flavonoids, carotenoids, and vitamin C contained in some FODMAP-rich vegetables (e.g., cauliflower, onion, garlic), or phenolic acid and anthocyanins present in fruits and blackberries. Wheat (which is excluded from the low-FODMAP diet) is a major source of phenolic acids, such as ferulic acid, caffeic acid, p-coumaric acid, p-hydroxybenzoic acid, vanillic acid, and protocatechuic acid [40]. Finally, the exclusion of dairy products in a low-FODMAP diet may favor a vitamin D deficiency [41].

We speculate that the nutritional risk of the low-FODMAP diet, in the long term, may follow an inverse socio-economic gradient, since persons with economical restraints may have limited access to many of the expensive, alternative dietary items included in the low-FODMAP diet (e.g., berries, exotic fruit, and pseudo-cereals).

## 5. Conclusions

The low-FODMAP diet can have a positive impact on the symptoms of IBS, particularly bloating and diarrhea. However, it remains to be proven whether this regimen is superior to conventional IBS diets. The drastic reduction of FODMAP intake could have physiological consequences on the colonocyte metabolism, the intestinal microbiota, and the nutritional status, which need further investigation. Based on our review, it might be helpful to consider the use of nutritional supplements to avoid possible deficiencies induced by a strict low-FODMAP diet over the long term.

## Figures and Tables

**Table 1:** Clinical trials on the effect of low-FODMAP diet in IBS patients.

First Author, Year	Patients	*n*	Study Design	Diet Duration	Results	Comment on the Study Design
Shepherd et al. [18]	Patients with IBS	*n* = 25	Low-FODMAP diet followed by DBPC crossover challenge with fructose and fructane	2 weeks	70% of patients receiving fructose, 77% receiving fructans, and 79% receiving a mixture reported symptoms were not adequately controlled, compared with 14% receiving glucose	Only some FODMAPs were tested in this study
Staudacher et al. [19]	Consecutive patients with IBS	I = 43 C = 39	Low FODMAP vs. standard IBS diet	9 months	Improved satisfaction and IBS score in I group	Lack of randomization
Staudacher et al. [20]	Patients with IBS	I = 19 C = 22	RCT, Low FODMAPs vs. habitual diet	1 weeks	More patients in the intervention group reported adequate control of symptoms (68%) compared with controls (23%)	Lack of blinding
Biesiekierski et al. [3]	Patients with NCGS and IBS	*n* = 37	Low-FODMAP diet followed by DBPC crossover challenge with gluten	3 weeks	Improvement with low FODMAP diet, no change between gluten and placebo challenge	Lack of control and no blinding during the low FODMAP diet
De Roest et al. [21]	consecutive patients with IBS	*n* = 90	Open, low FODMAP diet	16 months	Improvement of pre-study symptom	Lack of control group
Halmos et al. [22]	Patients with IBS and controls	I = 30 C = 8	Randomized, crossover, low-FODMAP diet vs. typical Australian diet	3 weeks	Lower overall gastrointestinal symptom scores while on a diet low in FODMAPs	Lack of blinding
Pedersen et al. [23]	Patients with IBS	I_1_ = 42 I_2_ = 41 C = 40	Randomized, controlled trial comparing the low FODMAP diet, treatment with Lactobacillus GG or a control diet	6 weeks	Both the low FODMAP diet and treatment with Lactobacillus GG were similarly effective	Lack of blinding
Chumpitazi et al. [24]	Children with IBS	*n* = 33	Randomized, double-blind, crossover trial, children with Rome III IBS completed a one-week baseline period. They then were randomised to a low FODMAP diet or typical American childhood diet	2 days	Less abdominal pain occurred during the low FODMAP diet vs. typical diet	Complete blinding unlikely. Short duration of challenge (two days)
Whigham et al. [25]	Patients with IBS	*n* = 365	Evaluation of low FODMAP diet administered in a dietitian-led group education or traditional one-to-one education	6 weeks	Significant decrease in symptom severity from baseline to follow-up for both groups but no difference in symptom response between group and one-to-one education	Lack of a control group; no randomization
Böhn et al. [26]	Patients with IBS	I = 33 C = 34	Multi-center, parallel, single-blind study. Subjects were randomly assigned to for four weeks to a low-FODMAP or standard IBS diet	4 weeks	The severity of IBS symptoms was reduced in both groups during the intervention in both groups before vs. at the end of the four-week diet, without a significant difference between the groups	Single blinding
McIntosh K et al. [27]	Patients with IBS	I = 19 C = 18	Controlled, single blind study with randomization to a low or high-FODMAP diet for three weeks	3 weeks	The IBS severity symptom score (SSS) was reduced in the low-FODMAP diet group but not the high-FODMAP group	Single blinding
Peters et al. [28]	Patients with IBS	I_1_ = 25 I_2_ = 24 I_3_ = 25	Consecutive patients were randomised to receive hypnotherapy, low-FODMAP diet or a combination	6 weeks	Improvements in overall symptoms were observed from baseline to week six for hypnotherapy, diet and combination with no difference across groups	No control group, no blinding
Laatikainen et al. [29]	Patients with IBS	*n* = 87	randomised double blind controlled cross-over study. Participants were supplied with both regular rye bread and low-FODMAP rye bread for four weeks	4 weeks	Many signs of IBS were milder on the low-FODMAP rye bread but no differences were detected in IBS-SSS or quality of life	Well-designed study; only rye FODMAPs were tested
Valeur et al. [30]	Patients with IBS	*n* = 63	Consecutive patients participating in a four-week FODMAP-restricted diet	4 weeks	Following the dietary intervention, IBS-SSS scores improved significantly	Lack of control group, and lack of blinding
Eswaran et al. [31]	Patients with IBS-D	I_1_ = 45 I_2_ = 39	Single-center, randomized-controlled trial comparing a low-FODMAP with the mNICE diet for four weeks.	4 weeks	40%–50% of patients reported adequate relief of their IBS-D symptoms with the low-FODMAP diet or a diet based on modified NICE guidelines. The low-FODMAP diet led to significantly greater improvement in individual IBS symptoms, particularly pain and bloating	Lack of blinding
Major et al. [32]	Patients with IBS	*n* = 58	Three-period, cross-over study with a single dose of high- or low-FODMAP drink	1 day	More patients reached the predefined symptom threshold after intake of inulin or fructose than glucose. Controls had lower symptom scores during the period after drink consumption, despite similar MRI parameters and breath hydrogen responses	Lack of blinding
Hustoft et al. [33]	Patients with IBS	*n* = 20	After three weeks of low-FODMAP patients were randomized and double-blindly assigned to receive a supplement of either FOS (FODMAP) or maltodextrin (placebo) for the next 10 days, followed by a three-week washout period before crossover	10 days	Irritable bowel syndrome symptoms consistently improved after three weeks of low FOMAP, and significantly more participants reported symptom relief in response to placebo than FOS	Only one type of FODMAP was investigated in this study

FODMAP: Fermentable Oligo-, Di- and Mono-saccharides And Polyols; IBS: Irritable bowel syndrome; IBS-D: Irritable bowel syndrome with diarrhea; RCT: randomized controlled trial; NCGS: non celiac gluten sensitivity; DBPC: double-blind placebo-controlled; I: intervention; C: control; SSS: severity symptom score; NICE: National Institute for Health and Care Excellence; FOS: fructo-oligosaccharides; GOS: galacto-oligosaccharides.

**Table 2:** Common food that need to be excluded from the low-FODMAP diet.

Food Type	To be Excluded (High-FODMAP Content)
Cereals and their derivatives	Wheat, barley, rye
Legumes	All (lentils, beans, chickpeas, soy, peas)
Vegetables	Artichokes, asparagus, cauliflower, garlic, leeks, mushrooms, onions, scallions, shallots, snow peas
Fruit	Apples, apricots, Asian pears, blackberries, cherries, figs, jackfruit, mangoes, nectarines, peaches, pears, persimmon, plums, prunes, tamarillo, watermelon, white peaches, grape
Dairy products	Regular milk, ice cream, soft cheeses, yogurt
